# Extraoral Versus Intraoral Photobiomodulation for the Prevention of Oral Mucositis in Pediatric Patients Undergoing High‐Dose Methotrexate Chemotherapy

**DOI:** 10.1111/scd.70279

**Published:** 2026-11-02

**Authors:** Mariana Lena Sassi, Nicoly Ghilardi Cardoso, Rafael Zancan Mobile, Bruna da Fonseca Wastner, Mara Albonei Dudeque Pianovski, Robinson Sabino‐Silva, Marco Guevara‐Vega, Douglas Carvalho Caixeta, Laurindo Moacir Sassi, Melissa Rodrigues de Araujo

**Affiliations:** ^1^ Department of Stomatology Federal University of Paraná (UFPR) Curitiba Paraná Brazil; ^2^ Pediatric Oncology Department Hospital Erastinho Curitiba Paraná Brazil; ^3^ Institute of Biomedical Sciences Federal University of Uberlândia (UFU) Uberlândia Minas Gerais Brazil; ^4^ Oncology Department Hospital Erasto Gaertner Curitiba Paraná Brazil

**Keywords:** methotrexate, oral mucositis, pediatric oncology, photobiomodulation therapy, preventive dentistry

## Abstract

**Aims:**

To compare intraoral (IO) and extraoral (EO) photobiomodulation therapy (PBMT) for preventing oral mucositis (OM) in pediatric patients receiving high‐dose methotrexate (HD‐MTX).

**Methods and Results:**

This prospective, open‐label, randomized clinical trial included 75 HD‐MTX treatment episodes from 31 pediatric patients aged 1–18 years. Each episode was randomized to IO‐PBMT or EO‐PBMT with red light at 660 nm. Preventive PBMT was initiated before HD‐MTX infusion and administered daily until discharge. OM was assessed at baseline and on days 3, 5, and 10 using the World Health Organization scale. Pain, salivary pH, and salivary biochemical profiles were secondary or exploratory outcomes. Cumulative OM occurred in 22/37 IO‐PBMT episodes (59.5%) and 12/38 EO‐PBMT episodes (31.6%). After accounting for repeated episodes within patients, OM risk was higher with IO‐PBMT (risk ratio, 1.84; 95% CI, 1.07–3.19; *p* = 0.029). Pain scores, salivary pH, and biochemical profiles did not differ significantly between groups. EO‐PBMT required substantially less application time, and no adverse effects were reported.

**Conclusion:**

EO‐PBMT was associated with lower cumulative OM occurrence, shorter application time, and good tolerability, supporting its use as a promising, less burdensome preventive strategy in pediatric oncology.

## 1 Introduction

Childhood cancer treatment commonly relies on multimodal therapeutic approaches, with chemotherapy representing a central component of care. Children undergoing antineoplastic therapy represent a vulnerable population within special care dentistry, as treatment‐related oral complications may impair oral function and complicate routine dental care [1].

Methotrexate (MTX) is widely incorporated into pediatric chemotherapy protocols and, particularly when administered at high doses, is associated with clinically relevant oral adverse effects. Among these, oral mucositis (OM) is one of the most significant complications in children receiving MTX‐based chemotherapy [2, 3, 4]. OM may adversely affect oral comfort, speech, swallowing, and nutritional intake [5]. The oral mucosa is particularly vulnerable to cytotoxic injury because of its rapid epithelial turnover [5].

OM is characterized by inflammatory injury and ulceration of the oral mucosa, resulting in pain, functional impairment, nutritional compromise, and increased infection risk [5, 6, 7]. Severe cases may negatively affect quality of life and contribute to chemotherapy dose reductions, treatment delays, prolonged hospitalization, and increased morbidity [5, 6, 7]. These consequences reinforce the importance of preventive oral care strategies in pediatric oncology.

Saliva plays an important role in maintaining oral mucosal homeostasis and may reflect systemic physiological and inflammatory changes [8]. Chemotherapy‐associated alterations in salivary parameters, oral microbiota, and inflammatory mediators have been investigated in relation to OM development and severity in pediatric oncology patients [9, 10]. Salivary analysis therefore represents a non‐invasive and patient‐centered approach that is particularly suitable for children with special health care needs, as it permits repeated and well‐tolerated biological monitoring [8, 9, 10].

Fourier‐transform infrared (FTIR) spectroscopy has been applied to saliva as a rapid and reagent‐free technique capable of detecting biochemical variations involving proteins, lipids, and carbohydrates and identifying disease‐related molecular signatures [11, 12]. From a special care dentistry perspective, FTIR‐based salivary analysis may represent a complementary and non‐invasive approach for exploring biochemical changes in pediatric patients undergoing chemotherapy, particularly in those with limited tolerance for invasive or repeated assessments.

Several preventive and therapeutic strategies have been proposed for the management of OM in pediatric oncology patients [6, 7]. Intraoral photobiomodulation therapy (IO‐PBMT) has demonstrated favorable effects in the prevention and management of chemotherapy‐induced OM in children [3, 13] and is supported by pediatric and MASCC/ISOO clinical practice guidelines [6, 7, 14]. Recent systematic reviews have further supported the preventive and therapeutic benefits of PBMT for chemotherapy‐induced OM, particularly in pediatric populations, while emphasizing the heterogeneity of irradiation parameters and the need for standardized clinical protocols [15, 16]. However, intraoral application may be challenging in pediatric patients because of discomfort, limited cooperation, and restricted access to posterior oral and oropharyngeal regions.

Extraoral photobiomodulation therapy (EO‐PBMT) has been proposed as an alternative delivery approach, with clinical studies reporting promising findings for OM prevention and management [17, 18, 19]. EO‐PBMT does not require prolonged mouth opening and may allow shorter and less burdensome treatment sessions, characteristics that are particularly relevant in pediatric care [17, 19, 20]. Prophylactic EO‐PBMT has been associated with favorable mucositis‐related and patient‐reported outcomes [18] while a pediatric feasibility study demonstrated that extraoral application was acceptable and well tolerated during hematopoietic cell transplantation [19]. Nevertheless, challenges remain regarding the standardization of irradiation parameters and the attenuation of light energy through extraoral tissues before reaching the oral mucosa [20, 21]. These knowledge gaps are especially relevant in pediatric oncology, where preventive oral care strategies must be both effective and clinically feasible.

Given the clinical relevance of minimizing oral complications in pediatric oncology patients with special health care needs, alternative PBMT delivery approaches warrant further investigation [6, 7]. Behavioral limitations, treatment‐related fatigue, pain, and reduced tolerance to oral procedures may hinder the implementation of conventional intraoral protocols in children [19]. In this context, EO‐PBMT may represent a more feasible and acceptable preventive approach because it does not require prolonged mouth opening and has been reported as well tolerated in oncology settings [17, 19, 20].

Therefore, the present study aimed to compare the preventive effectiveness of extraoral and intraoral PBMT for the prevention of OM in pediatric patients undergoing HD‐MTX chemotherapy. Secondary outcomes included the assessment of salivary pH and the exploratory characterization of salivary biochemical profiles using FTIR spectroscopy, with analysis of protein‐, lipid‐, and carbohydrate‐related spectral regions.

## 2 METHODS

### 2.1 Ethical Aspects and Study Design

This prospective, open‐label randomized clinical trial was conducted at XX Hospital a tertiary pediatric oncology center located in XX between February 2022 and June 2023. The study was approved by the Institutional Research Ethics Committee (CAAE: 52808121.1.0000.0098; approval number: 5.230.668) and was conducted in accordance with the Declaration of Helsinki. Written informed consent was obtained from the participants’ parents or legal guardians before enrollment. Assent was also obtained from children and adolescents when developmentally appropriate. Because the intervention routes and treatment durations were visibly different, blinding of participants and operators was not feasible. Allocation was not concealed, and outcome assessors were not blinded.

The analytical sample comprised 75 evaluable HD‐MTX treatment episodes contributed by 31 unique pediatric patients. Some patients contributed more than one eligible treatment episode during the study period. The unit of randomization was the treatment episode. Each eligible HD‐MTX treatment episode was independently randomized to the IO‐PBMT or EO‐PBMT group, including subsequent episodes contributed by the same patient. Consequently, an individual patient could contribute treatment episodes to either intervention group during the study period.

### 2.2 Participants and Sample Characteristics

A convenience sample of pediatric patients aged 1–18 years was recruited. Eligible patients had a diagnosis of leukemia, lymphoma, or osteosarcoma and were receiving HD‐MTX as part of institutional pediatric oncology protocols. Although the patients received multi‐agent chemotherapy regimens, the present study evaluated treatment episodes involving exclusively HD‐MTX administration.

The MTX dose was prescribed by the pediatric oncology team according to the disease‐specific chemotherapy protocol and was individually calculated based on each patient's body surface area. The total prescribed dose was calculated by multiplying the protocol‐defined dose, expressed in g/m^2^, by the patient's body surface area in m^2^. Patients with osteosarcoma received intravenous MTX at 12 g/m^2^ according to the GLATO protocol. Patients with acute lymphoblastic leukemia received intravenous MTX at 2.5 g/m^2^ according to the GBTLI protocol or 5 g/m^2^ according to the BFM 2009 protocol. Patients with lymphoma received intravenous MTX at 5 g/m^2^ according to the LNH 2016 protocol. All patients received hydration and supportive care according to institutional protocols during chemotherapy.

The planned sample was based on the number of eligible HD‐MTX treatment episodes expected at the institution, considering the patient volume during the year preceding study initiation. This pragmatic approach was adopted to ensure recruitment feasibility, and no formal power‐based sample size calculation was performed. A pilot phase was conducted to confirm the clinical applicability of the PBMT protocols.

Patients were assessed for eligibility during each hospitalization for HD‐MTX administration. Subsequent HD‐MTX treatment episodes from the same patient could be included, provided that all eligibility criteria were fulfilled at each admission. The inclusion criteria were age between 1 and 18 years, a diagnosis of leukemia, lymphoma, or osteosarcoma, and hospitalization for an eligible HD‐MTX treatment episode.

Treatment episodes involving oral infections or pre‐existing oral mucosal lesions at baseline were excluded. Episodes in which the PBMT protocol or the required study assessments were not completed were excluded from the final analysis.

### 2.3 Randomization and Intervention

Randomization was performed using a computer‐generated random sequence created with the Random.org platform. Each eligible treatment episode was allocated to either the IO‐PBMT group or the EO‐PBMT group. No allocation concealment was implemented.

Preventive PBMT was initiated before HD‐MTX infusion and was administered daily until hospital discharge, typically for 4–7 days, depending on MTX clearance. Device and irradiation parameters for both intervention groups are summarized in Table 1. The IO‐PBMT parameters were selected with reference to published pediatric protocols and clinical practice guidelines [3, 6, 7, 13, 14]. The EO‐PBMT protocol was based on previous clinical, feasibility, and dosimetric studies of extraoral delivery [17, 19, 20, 21].

**TABLE 1:** Parameters of intraoral and extraoral photobiomodulation therapy.

Parameter	IO‐PBMT	EO‐PBMT
PBMT source	AlGaInP diode laser	AlGaInP diode laser
Wavelength	660 nm	660 nm
Operating mode	Continuous wave	Continuous wave
Application technique	Contact	Contact
Beam area	0.098 cm^2^	4 central emitters: 0.029 cm^2^ 4 lateral emitters: 0.233 cm^2^
Power output/emitter	100 mW	100 mW^*^
Radiant exposure	10.02 J/cm^2^	4 central emitters: 34.50 J/cm^2^ 4 lateral emitters: 4.30 J/cm^2^
Energy per point/emitter	1 J	1 J
Number of irradiation sites/regions per session	35–41 points	3 regions (8 emitters per region)
Exposure duration per point/region	10 s	10 s^*^
Total energy per session	35–41 J	24 J

Abbreviations: AlGaInP, aluminum gallium indium phosphide; EO‐PBMT, extraoral photobiomodulation therapy; IO‐PBMT, intraoral photobiomodulation therapy; PBMT, photobiomodulation therapy.

* For treatment episodes involving patients with darker skin phototypes, the EO‐PBMT output power was reduced from 100 to 50 mW, and irradiation time was increased from 10 to 20 s to maintain an energy delivery of 1 J per emitter. The EO‐PBMT device contained eight emitters, delivering 8 J per treated region and 24 J per session.

### 2.4 IO‐PBMT

In the IO‐PBMT group, preventive PBMT was performed using an intraoral low‐level laser device (Therapy EC, DMC, São Carlos, São Paulo, Brazil). Irradiation was applied to 35–41 intraoral points, with the number of points adjusted according to patient age and oral cavity size. Irradiation sites included the bilateral buccal mucosa, lateral and ventral surfaces of the tongue, floor of the mouth, upper and lower lips, labial mucosa, and soft palate (Figure 1). The distribution of irradiation sites was based on previously published pediatric PBMT protocols [3, 13].

**FIGURE 1:**
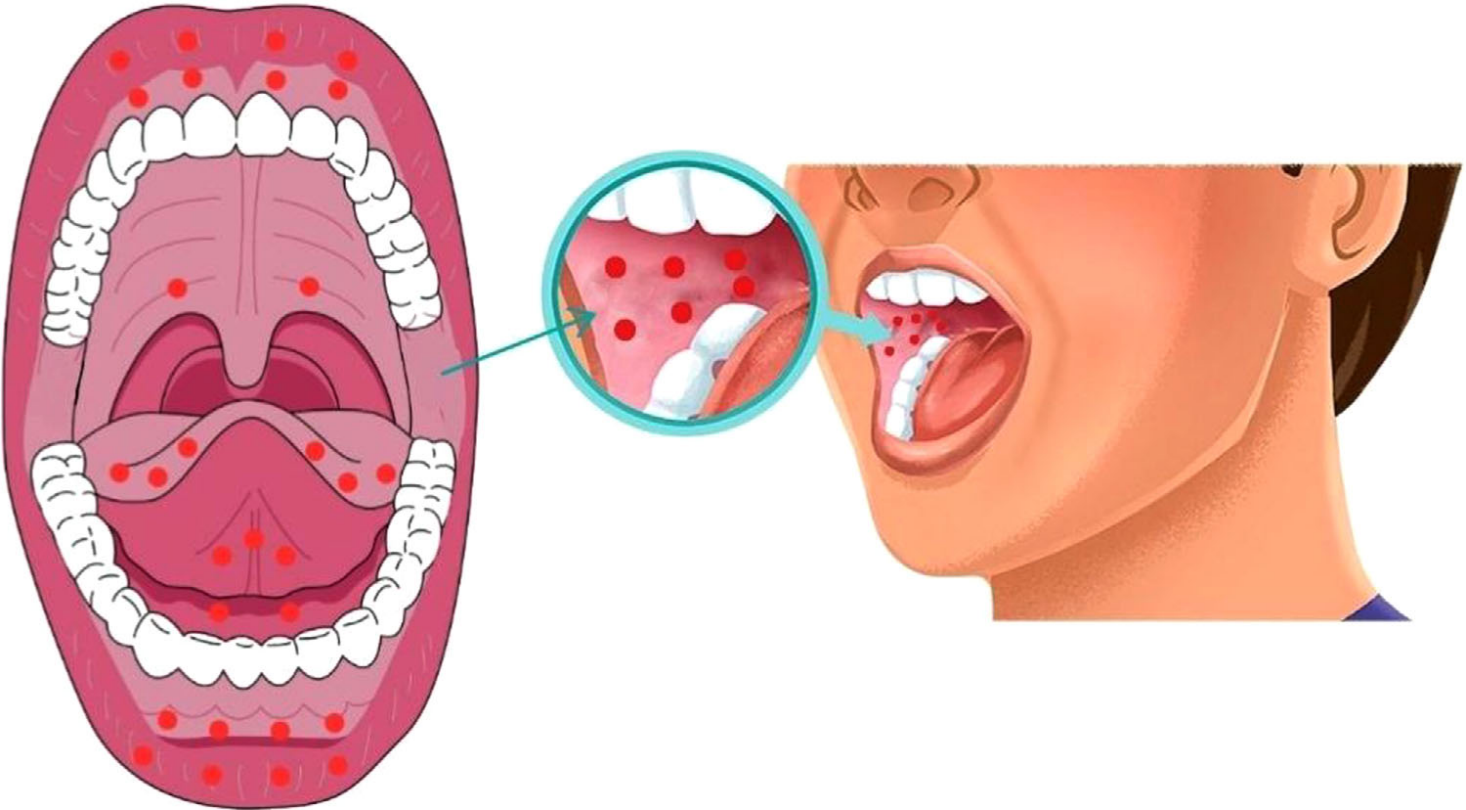
Intraoral photobiomodulation therapy protocol. Irradiation was applied to 35–41 intraoral points, according to the patient's age and oral cavity size, including the bilateral buccal mucosa, lateral and ventral surfaces of the tongue, floor of the mouth, upper and lower lips, labial mucosa, and soft palate.

### 2.5 EO‐PBMT

In the EO‐PBMT group, preventive PBMT was delivered using an extraoral device (E‐light RL, DMC, São Carlos, São Paulo, Brazil). Irradiation was applied in continuous‐wave mode to three extraoral regions: the right buccal region, left buccal region, and perioral orbicularis region. The device contained eight emitters, and each emitter delivered 1 J, corresponding to 8 J per treated region and a total energy of 24 J per session. During irradiation, the device was positioned perpendicular to and in direct contact with the patient's skin. The selection of the extraoral regions was informed by previous clinical and dosimetric investigations of EO‐PBMT [17, 19, 20, 21].

For participants with darker skin phototypes, output power was reduced from 100 to 50 mW as a protocol‐defined safety adjustment. Irradiation time was proportionally increased from 10 to 20 s to maintain an energy delivery of 1 J per emitter (Figure 2).

**FIGURE 2:**
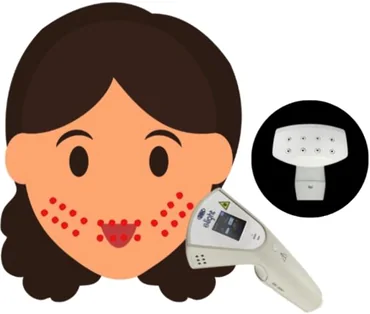
Extraoral photobiomodulation therapy protocol. Irradiation was applied to three treatment regions: the right buccal region, left buccal region, and perioral orbicularis region. The device contained eight emitters and delivered 1 J per emitter, corresponding to 8 J per treated region and a total energy of 24 J per session.

### 2.6 Data Collection and Clinical Assessment

Patients and their caregivers received standardized oral hygiene instructions throughout the study period. Demographic and clinical data, including sex, age, cancer diagnosis, and chemotherapy protocol, were extracted from medical records.

Clinical evaluations were performed at baseline (D0, before HD‐MTX infusion) and on days 3 (D3), 5 (D5), and 10 (D10) after infusion. For patients discharged before D10, the final clinical assessment and saliva collection were performed in person during outpatient follow‐up. Assessments were conducted by the same calibrated team responsible for the inpatient evaluations whenever feasible, and standardized assessment procedures were maintained throughout the study.

OM severity was assessed using the World Health Organization grading scale (grades 0–IV). Pain intensity was assessed using developmentally appropriate instruments: the Visual Analog Scale (VAS; 0–10) for children able to provide self‐reported scores and the Brazilian Faces Pain Scale described by Claro when use of the VAS was not developmentally appropriate, with caregiver assistance when necessary [22].

The primary outcome was the cumulative occurrence of OM during follow‐up, defined as the presence of OM at any assessment between D3 and D10. Secondary and exploratory outcomes included OM severity at each assessment, pain scores, salivary pH, and salivary biochemical profiles.

### 2.7 Saliva Collection and FTIR Analysis

Saliva samples were collected using the Salivette system (Sarstedt, Nümbrecht, Germany). Participants were instructed to keep the absorbent swab in the oral cavity for 1–2 min. Salivary pH was measured immediately using a digital pH meter (Phtek, PHS 3B), and the samples were subsequently stored at −80°C until analysis. Salivary biochemical analysis was performed using attenuated total reflection Fourier‐transform infrared (ATR‐FTIR) spectroscopy with a Vertex 70 spectrophotometer (Bruker Optics, Germany) equipped with a diamond ATR crystal. Spectra were recorded from 4000 to 400 cm^−^^1^ at a resolution of 4 cm^−^^1^ under controlled environmental conditions at 22°C–23°C. A standardized volume of 1 µL of saliva was air‐dried directly on the ATR crystal for 6 min before spectral acquisition, following a previously published salivary ATR‐FTIR methodology [11]. The spectral regions of interest included lipids (3000–2800 cm^−^^1^), proteins (1720–1470 cm^−^^1^), and carbohydrates (1200–910 cm^−^^1^) [11, 12].

### 2.8 Examiner Calibration

PBMT applications and clinical assessments were performed by a calibrated team consisting of two experienced dentists and one trained undergraduate student. Before study initiation, calibration and standardization sessions were conducted for device operation, WHO mucositis grading, saliva collection, and pain assessment. Whenever feasible, the same examiner followed each patient throughout the study to improve consistency.

### 2.9 Statistical Analysis

Statistical analyses were performed using JAMOVI version 2.3, except for the generalized estimating equation (GEE) analysis, which was conducted in Python version 3.13.5 using the statsmodels package version 0.14.6. Data distribution was assessed using the Shapiro–Wilk test. All tests were two‐sided, with statistical significance set at *p* < 0.05.

Baseline age was compared using the Mann–Whitney *U* test, whereas categorical variables were analyzed using Pearson's chi‐square, Fisher's exact, or Fisher–Freeman–Halton exact tests, as appropriate. The primary outcome was analyzed using a Poisson GEE model with a log link, exchangeable correlation structure, robust standard errors, and patient identification as the clustering variable. Results were expressed as risk ratios with 95% confidence intervals.

OM severity was summarized descriptively. Application time and pain scores were compared using the Mann–Whitney *U* test, salivary pH using Student's *t*‐test, and FTIR outcomes using Student's *t*‐test in GraphPad Prism version 8.0. Secondary and exploratory analyses were conducted at the treatment‐episode level. Salivary analyses were based on the samples available at each assessment time point, and missing salivary values were not imputed.

## 3 Results

### 3.1 Study Characteristics and Sample Demographics

Of the 80 HD‐MTX treatment episodes initially randomized, five were excluded from the final analysis because the required study data could not be obtained: three from the IO‐PBMT group and two from the EO‐PBMT group. The final analysis included 75 evaluable treatment episodes of 31 unique pediatric patients, comprising 37 IO‐PBMT episodes and 38 EO‐PBMT episodes.

The treatment episodes involved patients aged 1–18 years. The median age was 8.0 years (IQR, 6.0–12.0) in the IO‐PBMT group and 5.5 years (IQR, 4.0–8.0) in the EO‐PBMT group. No statistically significant between‐group differences were detected regarding age, sex, skin color, or underlying malignancy. Sociodemographic and clinical characteristics are summarized in Table 2.

**TABLE 2:** Sociodemographic and clinical characteristics of the evaluable HD‐MTX treatment episodes.

Characteristic	IO‐PBMT(*n* = 37)	EO‐PBMT(*n* = 38)	Total(*N* = 75)	*p*‐value
**Age, years, median (IQR)**	8.0 (6.0–12.0)	5.5 (4.0–8.0)	7.0 (5.0–12.0)	0.057
**Sex, n (%)**				0.535
Female	13 (35.1)	16 (42.1)	29 (38.7)	—
Male	24 (64.9)	22 (57.9)	46 (61.3)	—
**Skin color, n (%)**				1.000
White	34 (91.9)	35 (92.1)	69 (92.0)	—
Brown	3 (8.1)	3 (7.9)	6 (8.0)	—
**Tumor type, n (%)**				0.459
Leukemia	25 (67.6)	29 (76.3)	54 (72.0)	—
Osteosarcoma	9 (24.3)	5 (13.2)	14 (18.7)	—
Lymphoma	3 (8.1)	4 (10.5)	7 (9.3)	—

*Note*: Data are presented as number (%) unless otherwise indicated. Age is presented as median and interquartile range (IQR). Data refer to treatment episodes; the 75 evaluable episodes were contributed by 31 unique pediatric patients, and some patients contributed more than one episode. Age was compared using the Mann–Whitney *U* test. Sex was compared using Pearson's chi‐square test. Skin color was compared using Fisher's exact test, and tumor type was compared using the Fisher–Freeman–Halton exact test. A single overall ***p***‐value was calculated for each variable.

Abbreviations: EO‐PBMT, extraoral photobiomodulation therapy; HD‐MTX, high‐dose methotrexate; IO‐PBMT, intraoral photobiomodulation therapy; IQR, interquartile range.

In the EO‐PBMT group, three treatment episodes involving patients with darker skin phototypes were submitted to irradiation with reduced power (50 mW) to minimize melanin‐related light absorption and potential thermal discomfort. To maintain the same delivered energy (1 J per emitter), irradiation time was proportionally increased from 10 to 20 s. This adjustment was implemented to preserve dose consistency while ensuring patient comfort and safety. Both PBMT protocols were well tolerated, and no adverse effects were reported. The mean application time was approximately 30 s per session for EO‐PBMT. IO‐PBMT required approximately 15–20 min of active irradiation, while the total session duration ranged from 30 to 60 min, depending on patient cooperation.

### 3.2 OM Outcomes

Cumulative OM occurred in 22 of 37 IO‐PBMT treatment episodes (59.5%) and in 12 of 38 EO‐PBMT treatment episodes (31.6%). After accounting for repeated treatment episodes within patients using a GEE model, the risk of OM was higher in the IO‐PBMT group than in the EO‐PBMT group (risk ratio, 1.84; 95% CI, 1.07–3.19; *p* = 0.029). No cases of OM were observed at baseline (D0) in either group. When OM occurrence was analyzed according to individual evaluation time points (D3, D5, and D10), no statistically significant differences were detected between groups (*p* > 0.05). The distribution of OM grades at each assessment time point is presented in Table 3.

**TABLE 3:** Distribution of oral mucositis grades in the IO‐PBMT and EO‐PBMT groups at each assessment time point.

OM grade	IO‐PBMT				EO‐PBMT			
	D0	D3	D5	D10	D0	D3	D5	D10
**0**	37 (100%)	34 (91.9%)	28 (75.7%)	22 (59.5%)	38 (100%)	38 (100%)	36 (94.7%)	28 (73.7%)
**I**	0	2 (5.4%)	3 (8.1%)	2 (5.4%)	0	0	2 (5.3%)	3 (7.9%)
**II**	0	1 (2.7%)	6 (16.2%)	7 (18.9%)	0	0	0	3 (7.9%)
**III**	0	0	0	6 (16.2%)	0	0	0	3 (7.9%)
**IV**	0	0	0	0	0	0	0	1 (2.6%)
**Total**	**37 (100%)**	**37 (100%)**	**37 (100%)**	**37 (100%)**	**38 (100%)**	**38 (100%)**	**38 (100%)**	**38 (100%)**

*Note*: Values are presented as number (%). Data refer to treatment episodes. The cumulative occurrence of OM between D3 and D10 was observed in 22 of 37 IO‐PBMT episodes (59.5%) and 12 of 38 EO‐PBMT episodes (31.6%). After accounting for repeated treatment episodes within patients using a generalized estimating equation model, the risk ratio was 1.84 (95% CI, 1.07–3.19; *p* = 0.029). Each episode was counted only once in the cumulative outcome, regardless of the number of assessments at which OM was present.

Abbreviations: D0, baseline; D3, day 3; D5, day 5; D10, day 10; EO‐PBMT, extraoral photobiomodulation therapy; IO‐PBMT, intraoral photobiomodulation therapy; OM, oral mucositis.

Regarding severity, OM was categorized as absent (grade 0), mild (grades I–II), or moderate/severe (grades III–IV). At D3, mild OM was observed in 3 of 37 IO‐PBMT treatment episodes (8.1%), while no cases occurred in the EO‐PBMT group. At D5, mild OM was observed in 9 of 37 IO‐PBMT episodes (24.3%) and in 2 of 38 EO‐PBMT episodes (5.3%); no grade III–IV OM was observed in either group. At D10, mild OM was observed in 9 of 37 IO‐PBMT episodes (24.3%) and in 6 of 38 EO‐PBMT episodes (15.8%), whereas moderate/severe OM occurred in 6 of 37 IO‐PBMT episodes (16.2%) and in 4 of 38 EO‐PBMT episodes (10.5%).

### 3.3 Pain Assessment

Pain intensity was assessed at all evaluation time points (D0, D3, D5, and D10). Given the wide age range of participants, pain assessment was adapted to the patients’ developmental stage. For children able to provide self‐reported scores, a VAS (0–10) was used, whereas the Brazilian Faces Pain Scale described by Claro was used when the VAS was not developmentally appropriate, with caregiver assistance when necessary.

Maximum pain scores ranged from 6 at D3 to 10 at D10 in the IO‐PBMT group and from 3 at D3 to 10 at D10 in the EO‐PBMT group. Despite these isolated high scores, mean pain scores remained close to zero throughout follow‐up in both groups. No statistically significant differences in pain scores were observed between the groups at any individual evaluation time point (all *p* > 0.05), indicating no evidence of differential pain outcomes between the two PBMT modalities.

### 3.4 Salivary pH and FTIR Analysis (Exploratory Outcomes)

Salivary pH remained close to neutrality throughout follow‐up, with no statistically significant differences between groups at any evaluation time point (all *p* > 0.05) (Table 4). Salivary biochemical profiles were analyzed as exploratory outcomes using FTIR spectroscopy. The average FTIR spectra obtained at D0, D3, D5, and D10 showed similar patterns in the two groups. Quantitative analysis of spectral bands corresponding to lipids (3000–2800 cm^−^^1^), proteins (1720–1470 cm^−^^1^), and carbohydrates (1200–910 cm^−^^1^) revealed no statistically significant between‐group differences at any evaluation time point (all *p* > 0.05). No differential changes in the evaluated salivary biochemical regions were detected between the IO‐PBMT and EO‐PBMT groups during follow‐up.

**TABLE 4:** Salivary pH in the IO‐PBMT and EO‐PBMT groups at D0, D3, D5, and D10.

Assessment time	Group	Mean	Standard deviation	*p*‐value
D0	IO‐PBMT	7.16	0.676	0.076
	EO‐PBMT	6.74	0.855	
D3	IO‐PBMT	6.72	0.801	0.507
	EO‐PBMT	6.90	0.860	
D5	IO‐PBMT	7.27	0.529	0.062
	EO‐PBMT	6.67	0.839	
D10	IO‐PBMT	6.94	0.564	0.099
	EO‐PBMT	7.55	0.406	

*Note*: Values are presented as mean and standard deviation. Between‐group comparisons at each assessment time point were performed using Student's *t*‐test. Salivary outcomes were exploratory and were analyzed at the treatment‐episode level.

Abbreviations: D0, baseline; D3, day 3; D5, day 5; D10, day 10; EO‐PBMT, extraoral photobiomodulation therapy; IO‐PBMT, intraoral photobiomodulation therapy.

## 4 Discussion

The primary finding of this randomized clinical trial, which included 75 HD‐MTX treatment episodes of 31 unique pediatric patients, was a substantially lower cumulative occurrence of OM in the EO‐PBMT group than in the IO‐PBMT group during follow‐up (31.6% vs. 59.5%). This difference remained statistically significant after accounting for repeated treatment episodes within patients (risk ratio, 1.84; 95% CI, 1.07–3.19; *p* = 0.029). This clinically relevant difference supports EO‐PBMT as a promising preventive approach for pediatric patients undergoing HD‐MTX chemotherapy. EO‐PBMT was also well tolerated, required considerably less application time, and was not associated with adverse effects, highlighting its practical value in pediatric oncology and special care dentistry.

These findings do not diminish the established role of IO‐PBMT, which remains supported by pediatric and MASCC/ISOO clinical practice guidelines [6, 7, 14]. Rather, they indicate that EO‐PBMT may provide an effective and less burdensome alternative, particularly for children in whom fatigue, discomfort, limited cooperation, or difficulty maintaining mouth opening can complicate prolonged intraoral treatment.

Although IO‐PBMT is well established, clinical evidence regarding EO‐PBMT remains comparatively limited and has primarily involved patients undergoing hematopoietic stem cell transplantation or treatment for head and neck cancer [17, 18, 19]. In patients undergoing hematopoietic stem cell transplantation, EO‐PBMT has shown clinical outcomes comparable to those of intraoral application, with the additional advantage of shorter treatment sessions [17]. Prophylactic EO‐PBMT has also been associated with delayed OM onset, reduced pain, and decreased analgesic and anti‐inflammatory medication requirements in adults undergoing head and neck cancer treatment [18]. In pediatric hematopoietic cell transplantation, daily extraoral application has been reported as acceptable, well‐tolerated, and clinically feasible [19].

The present study extends this evidence by directly comparing EO‐ and IO‐PBMT in pediatric treatment involving HD‐MTX administration. This setting is clinically relevant because OM may cause pain, nutritional impairment, increased infection risk, prolonged hospitalization, and interruption or modification of antineoplastic treatment [5, 6, 7]. The lower cumulative occurrence observed with EO‐PBMT therefore represents an important clinical finding in a population in which preventive strategies must combine effectiveness, tolerability, and ease of application.

Previous pediatric clinical studies and evidence syntheses have reported favorable effects of PBMT on chemotherapy‐induced OM, including reductions in occurrence, severity, duration, and associated pain [3, 13, 15, 16, 23, 24, 25, 26, 27]. Nevertheless, controlled evidence specifically evaluating the extraoral route in pediatric patients remains scarce. The present findings, therefore, contribute new head‐to‐head clinical evidence supporting EO‐PBMT as a feasible preventive modality during HD‐MTX chemotherapy.

A distinctive feature of this study was the exclusive use of red light at 660 nm, a wavelength previously employed in pediatric intraoral PBMT protocols [3, 13]. Published extraoral protocols, in contrast, have used heterogeneous irradiation parameters, including infrared wavelengths and combinations of red and infrared light [17, 18, 19, 20, 21]. Despite this variability, the favorable clinical outcome observed in the present study suggests that red‐light EO‐PBMT may be a viable approach for OM prevention in pediatric oncology.

Few clinical investigations have directly compared EO‐ and IO‐PBMT. A previous randomized study involving patients undergoing hematopoietic stem cell transplantation found no significant difference in OM outcomes between the modalities and concluded that EO‐PBMT was as effective as IO‐PBMT while requiring shorter treatment sessions [17]. In the present study, EO‐PBMT was associated with a lower cumulative occurrence of OM. The substantially shorter extraoral sessions may have reduced treatment interruptions or incomplete irradiation in children with limited cooperation. In addition, extraoral application distributes light across a broader transcutaneous field and may reach deeper tissue compartments. However, treatment fidelity, the proportion of the planned intraoral dose effectively delivered, and the effective light dose reaching the oral mucosa were not directly measured.

The biological effects of PBMT have been associated with the absorption of red or near‐infrared light by cellular chromophores, including cytochrome c oxidase. This interaction may modulate mitochondrial activity, ATP production, reactive oxygen species, inflammatory signaling, and pathways involved in tissue repair. PBMT may consequently reduce inflammation, promote tissue healing, and contribute to analgesic responses [28]. These mechanisms provide biological plausibility for the favorable clinical effects observed with PBMT, although the present study was not designed to investigate molecular mechanisms directly.

No statistically significant differences in pain scores were observed between IO‐ and EO‐PBMT at any evaluation time point. Mean pain scores remained low throughout follow‐up in both groups, and the highest scores were recorded in only two treatment episodes involving severe OM. These findings are consistent with pediatric clinical studies and evidence syntheses reporting favorable effects of PBMT on OM‐related pain [23, 25, 26, 27].

Pain assessment was adapted to developmental stage, using the VAS in children able to provide self‐reported scores and the Brazilian Faces Pain Scale described by Claro when the VAS was not developmentally appropriate, with caregiver assistance when necessary [22]. Because analgesic consumption was not systematically compared between groups, future studies should include this variable as a complementary outcome when investigating PBMT‐related pain control. The FLACC scale was not used in the present study; future studies should consider validated observational instruments, such as the FLACC scale, for pain assessment in preverbal or non‐communicative pediatric patients [29].

Saliva and the oral microbiota may influence oral mucosal homeostasis during chemotherapy [8, 9, 10], while basic oral care remains an important component of supportive pediatric oncology care [6, 7]. In the present study, all participants received standardized oral hygiene instructions. Salivary pH remained close to neutrality throughout follow‐up, with no statistically significant differences between IO‐ and EO‐PBMT. ATR‐FTIR spectroscopy likewise revealed comparable biochemical profiles between groups, with no differential changes detected in the evaluated lipid, protein, or carbohydrate spectral regions [11, 12]. These findings suggest that the two PBMT modalities produced similar salivary biochemical profiles under the analytical conditions employed. As an exploratory assessment, the FTIR analysis broadens the characterization of the clinical response and supports further investigation of saliva‐based monitoring in pediatric oncology.

EO‐PBMT demonstrated important practical advantages. Its application was non‐invasive, did not require sustained mouth opening, and required approximately 30 s per session, compared with 15–20 min of active irradiation for IO‐PBMT. Previous studies have similarly emphasized the feasibility and shorter duration of extraoral treatment sessions [17, 19]. These characteristics may improve patient tolerance, treatment adherence, and workflow efficiency, particularly among fatigued, medically fragile, or poorly cooperative pediatric patients. Proper device calibration and dosimetric control remain important for clinical implementation of EO‐PBMT. Because light must pass through skin, subcutaneous tissue, and muscle before reaching the oral mucosa, tissue‐related attenuation may influence the effective dose delivered to the target region [20, 21]. Nevertheless, the favorable clinical results and absence of adverse effects observed in the present study support the feasibility of the protocol employed.

Although no statistically significant between‐group differences were observed at D3, D5, and D10 evaluation time points, the cumulative occurrence of OM during D3–D10 was the predefined primary outcome and captured whether mucositis developed at any point during follow‐up. The favorable cumulative result, therefore, remains clinically meaningful, while the individual time‐point findings provide complementary information regarding the temporal distribution of OM.

Some methodological considerations should be acknowledged. This was a single‐center study based on a convenience sample, and no formal power‐based sample size calculation was performed. Although outcome assessors were not blinded, standardized assessment procedures and calibrated examiners were used throughout the study. Some patients contributed more than one eligible HD‐MTX treatment episode; however, within‐patient correlation was addressed in the primary outcome analysis using a GEE model. Secondary and exploratory outcomes were analyzed at the treatment‐episode level and should therefore be interpreted accordingly. Although participants were treated according to different multi‐agent chemotherapy protocols, all treatment episodes evaluated in the present study included exclusively HD‐MTX. Despite these considerations, the prospective randomized design, standardized protocols, direct comparison of two clinically applicable interventions, and clustered analysis of the primary outcome strengthen the reliability and clinical relevance of the findings.

The study also had important strengths, including its prospective randomized design, direct comparison of two clinically applicable PBMT protocols, standardized treatment procedures, calibrated examiners, predefined assessment time points, consistent supportive care, and evaluation of both clinical and exploratory salivary outcomes. The use of a standardized WHO grading scale at predefined evaluation times is also relevant, given the heterogeneity of OM assessment instruments reported in pediatric oncology studies [30]. The inclusion of a broad pediatric age range reflects the practical challenges encountered in pediatric oncology and special care dentistry.

Future studies should investigate whether combined intraoral and extraoral PBMT protocols provide additional benefits in high‐risk pediatric oncology populations. A combined approach has previously been investigated in pediatric patients undergoing hematopoietic stem cell transplantation, providing clinical rationale for further evaluation of hybrid preventive protocols [31]. Multicenter randomized trials with formal sample size calculations, blinded outcome assessment, statistical models accounting for repeated treatment episodes and longitudinal measurements across all outcomes, and standardized extraoral dosimetry will be important for confirming the present findings and supporting broader clinical implementation.

Overall, EO‐PBMT was associated with a substantially lower cumulative occurrence of OM, markedly shorter application time, good treatment tolerance, and no reported adverse effects. These findings support EO‐PBMT as a clinically promising, feasible, and less burdensome preventive strategy for pediatric patients undergoing HD‐MTX chemotherapy.

## 5 Conclusion

Extraoral PBMT was associated with a significantly lower cumulative occurrence of OM than intraoral PBMT in pediatric patients undergoing high‐dose MTX chemotherapy. No significant between‐group differences were observed in pain scores, salivary pH, or salivary biochemical profiles. Given its non‐invasive nature, substantially shorter application time, good tolerability, and clinical feasibility, EO‐PBMT may represent a promising preventive strategy for OM in pediatric oncology and may facilitate the implementation of supportive oral care protocols in this population.

## Author Contributions

**Melissa Rodrigues de Araujo**: conceptualization, methodology, investigation, data curation, formal analysis, writing – original draft, writing – review and editing, supervision, project administration. **Mariana Lena Sassi** and **Nicoly Ghilardi Cardoso**: responsible for patient recruitment, clinical procedures, data collection, and investigation. **Rafael Zancan Mobile**: formal analysis, statistical analysis, writing, data curation. **Robinson Sabino‐Silva, Marco Guevara‐Vega**, and **Douglas Carvalho Caixeta**: investigation, resources, data curation. **Bruna Wastner, Mara Albonei Dudeque Pianovski,** and **Laurindo Moacir Sassi**: methodology, validation, writing – review and editing. All authors have read and agreed to the published version of the manuscript.

## Ethics Statement

This study was approved by Erasto Gaertner Hospital Research Ethics Committee (Protocol No. 4.866.526) and conducted in accordance with the Declaration of Helsinki.

## Conflicts of Interest

All authors declare no conflicts of interest.
